# Comparative Hydrophobicity Analysis of Human β-Tubulin and Bacterial FtsZ Using the Fuzzy Oil Drop Model: Potential Implications for Chaperone-Assisted Folding

**DOI:** 10.34133/csbj.0250

**Published:** 2026-10-30

**Authors:** Irena Roterman, Katarzyna Stapor, Dawid Dulak, Leszek Konieczny

**Affiliations:** ^1^ Department of Bioinformatics and Telemedicine, Jagiellonian University—Medical College, 30-688 Kraków, Poland.; ^2^ Faculty of Automatic, Electronics and Computer Science, Department of Applied Informatics, Silesian University of Technology, 44-100 Gliwice, Poland.; ^3^ ASSA ABLOY Opening Solutions, Leszno, Poland.; ^4^ Chair of Medical Biochemistry, Jagiellonian University—Medical College, 31-034 Kraków, Poland.

## Abstract

The issue of protein folding *in vivo* remains an unsolved problem despite important progress achieved by artificial intelligence (AlphaFold). Experimental studies have shown that there are several mechanisms involved in this process. The main distinction concerns the involvement of chaperone proteins in this process as opposed to spontaneous folding. Evolutionary analysis shows that tubulin in eukaryotic organisms requires engagement of the ATP-dependent ring-shaped chaperonin TRiC/CCT, while the homologous protein in prokaryotic organisms obtains its functional structure without the support from other proteins. This study—limited to 2 examples: one human tubulin β chain and one structure of homologous bacterial FtsZ protein—presents hypothetical interpretation of these differences based on the external conditions for folding process. The differences of the distribution of hydrophobicity in the compared proteins are taken as the criteria for comparison. The fuzzy oil drop model (FOD-M) assumes the folding of prokaryotic proteins as a spontaneous process aided only by the presence of the water environment that directs the folding toward the generation of a centrally located hydrophobic core with a polar surface. Eukaryotic protein—tubulin—exhibits a distribution of hydrophobicity that lacks this type of order as it is interpreted based on the FOD-M assumptions. The difference seems to be the result of the involvement of different external force fields in the folding process. These 2 proteins are an example that suggest the need to include the active influence of the local environment. The hypothesis for *in silico* methods of folding process simulation is assumed to use a multiple-object optimization technique that takes into account the optimization of the energy state of protein E as a function dependent on 2 functions: *E*_INT_ (internal force field) and *E*_EXT_ (external force field).

## Introduction

The aim of a correctly performed protein folding process is to build a biologically active component that fulfils a strictly defined task in the functioning of a cell or an entire complex organism. Obtaining an incorrect form results in the absence of a tool to perform a specific task, as exemplified by diseases arising from the emergence of mutations [1]. Additionally, if an incorrectly formed structure leads to the creation of complexes, including those of significant size, this phenomenon may manifest itself in the form of neurodegenerative diseases [2–5]. Amyloids—fibril-like poly-complexes composed of proteins with a significantly altered structure compared to their active forms—are not always associated with the presence of mutations. This proves that the misfolding process is physical in nature and that the main cause of structural changes is the influence of the local environment [6]. It has been demonstrated that almost any protein can undergo amyloid transformation *in vitro* under appropriate conditions without resorting to the introduction of mutations [7]. The physical process of shaking leads to structural changes resulting in the formation of amyloid fibrils [8]. Shaking, a process that increases the presence of the air/water interface, is an external factor treated as the introduction of a new form of external force field. The assumption underlying the FOD-M model used in this study concerns the participation of an external force field actively influencing the folding process [9].

Spontaneous, reversible folding of proteins in the water environment is the result of the active presence of the water environment directing the folding process, similar to the micellization process. It involves generating a centrally located hydrophobic core isolated from the surrounding water by a polar surface obtained by exposing polar residues. A different effect is obtained when folding a polypeptide chain, e.g., in the cell membrane environment, where the expectation is the opposite: exposure of nonpolar residues and concentration of polar residues in the center (especially in the case of a protein acting as an ion channel). These 2 examples clearly reveal the influence of the environment (external force field) on the final folding, with an obvious need to optimize the internal force field resulting from nonbinding interactions between the components of the polypeptide chain.

The presence of chaperonins or chaperones, including prefoldin, which is required for proper protein folding, aims to prevent the influence of the water environment by introducing a specific external force field represented by these proteins. The role of chaperonins in the context of proteostasis is discussed in [10,11]. The relation between chaperonins and ribosome appears important in the context of protein synthesis [12]. The phenomenon of misfolding is significantly important with respect to misfolding-based diseases [13]. The detailed analysis of CCT/TRiC (chaperonin containing TCP-1/T-complex protein ring complex), Hsp90, and Hsp70 is presented in [14–17]. The intramolecular chaperone-mediated protein folding [18] appears important for general interpretation of the system of supporting proteins in protein synthesis processes [19–21]. The isolation in form of capsulation and in consequence folding in the chambers is treated as limitation of major kinetic complications and multimolecular association are prevented [22].

The FOD-M model takes into account the presence of support proteins as a factor altering the form of the external force field, creating a specific environment for the protein folding process.

The subject of this analysis is 2 proteins that perform a similar function in a eukaryotic and a prokaryotic cell. This protein is human tubulin, whose folding process requires the presence of both prefoldin and chaperonin: adenosine triphosphate (ATP)-dependent ring-shaped chaperonin TRiC/CCT. A prokaryotic protein identified as homologous to the FtsZ group, which obtains a similar structure in the interpretation of the super-secondary structure, does not require the participation of other proteins during folding. The folding process of this protein is spontaneous in the water environment. In light of FOD-M (the fuzzy oil drop model), this means the exclusive participation of the water environment.

## Materials and Methods

### Data

The analysis of the proteins discussed herein was based on reports discussed in [23]. The aforementioned work [23] states that: “While eukaryotic tubulin folding exhibits an obligate requirement for TRiC, its prokaryotic homologue FtsZ can spontaneously fold without chaperones”, referring to the articles [24,25].

The comparative analysis of proteins (suggested in [23]) includes human tubulin in the form of a dimer composed of α and β chains [Protein Data Bank (PDB) ID: 6I2I—only the β chain is analyzed [26]] and a prokaryotic protein from the FtsZ group (PDB ID: 1FSZ [27]) identified as homologous to tubulin [23].

### Programs used

The visualization of the 3-dimensional (3D) structures of the proteins in question was obtained using the VMD software [28,29].

The RD (relative distance) and *K* parameters were calculated using the HPHOB software available in the open access system (https://hphob.sano.science/) and a program enabling calculation of RD accessible on the GitHub platform:

https://github.com/KatarzynaStapor/FODmodel after obtaining a user account.

### FOD-M model for assessing hydrophobicity distribution

The main assumption of the fuzzy oil drop model is based on the assessment of amino acids as bipolar molecules with varying proportions of polarity and nonpolarity. In the water environment, bipolar molecules spontaneously form an arrangement called a micelle—an arrangement with centrally located nonpolar fragments and a polar surface that facilitates contact with the water environment. The original concept expressed by Kauzmann [30] treated the protein structure as an oil drop model. The modification involves replacing the discrete 2-layer (polar surface and hydrophobic core) distribution of hydrophobicity in the oil drop model with a continuous distribution with a gradual decrease in hydrophobicity levels as the distance from the center of the arrangement increases, reaching zero at the surface. This distribution is expressed by a 3D Gaussian function spread across the body of a given arrangement. The corresponding function is written as follows:

$$\begin{array}{c} H_{i}^{T}=\frac{1}{H_{\mathrm{sum}}^{T}}\text{exp}\left(\frac{-{\left(x_{i}-\bar{x}\right)}^{2}}{2\sigma_{x}^{2}}\right)\text{exp}\left(\frac{-{\left(y_{i}-\bar{y}\right)}^{2}}{2\sigma_{y}^{2}}\right)\text{exp}\left(\frac{-{\left(z_{i}-\bar{z}\right)}^{2}}{2\sigma_{z}^{2}}\right) \end{array}$$ (1)

where the values of the parameters σ_x_, σ_y_, and σ_z_ denote the size of the spherical micelle. In the case of proteins, the resulting structure is not necessarily spherical, which is why the values of these parameters differ for the respective directions, thus determining the size and shape of the molecule. The *x*, *y*, and *z* positions are the coordinates of the center of the coordinate system. Introducing the appropriate orientation of the protein molecule in the coordinate system, the geometric center of the molecule is located at the origin of the coordinate system (0,0,0), which means that the *X*, *Y*, and *Z* values are equal to 0.0. *x_i_*, *y_i_*, and *z_i_* denote the positions of effective atoms (averaged position of atoms belonging to particular amino acid). The distribution of hydrophobicity described by Eq. ((1) represents an idealized distribution—called T (theoretical), which serves as the reference distribution in the FOD-M model. It represents a centrally located maximum hydrophobicity with gradually decreasing levels as one moves away from the center, reaching zero (zero hydrophobicity corresponds to polar levels) at the surface. The example of T distribution in form limited to 1D (for clarity) is presented in Fig. 1A (blue line).

**Fig. 1.**
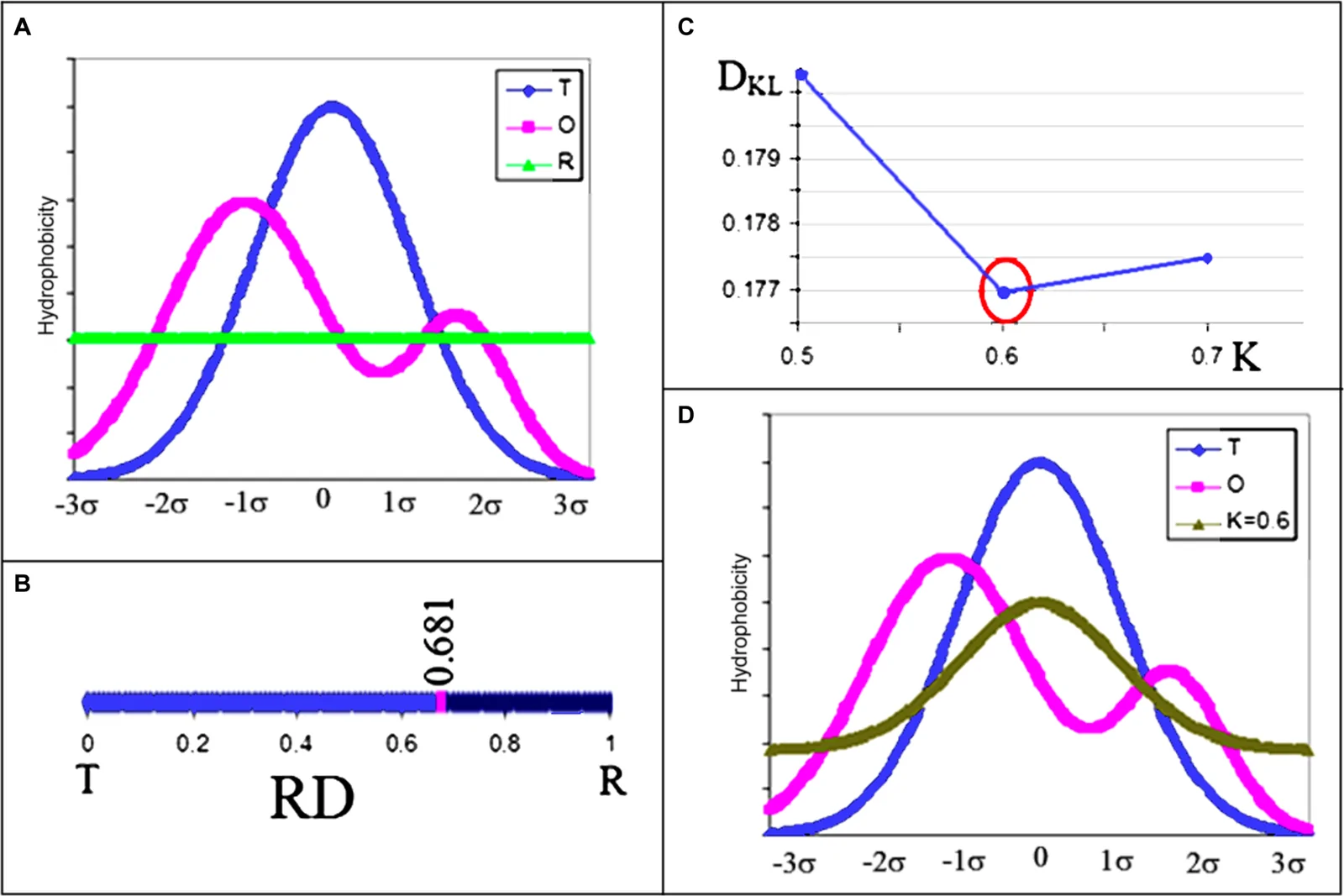
Graphical representation of the FOD-M model (reduced to a 1D representation for clarity). (A) Distribution T (blue) [Eq. ((1)], O (pink) [Eq. (2)], and R (green) expressing *R*_i_ = 1/*N*, where *N* is the number of amino acids in chain (protein). (B) RD value as an example shown in (A) [calculated according to Eq. ((4)]. (C) Searching for the minimum value of D_KL_(O|M) for the example given in (A) [according to Eq. (6) for different *K* values to find the minimum of the D_KL_(O|M) value]. (D) Profile T [blue, as in (A)], O [pink, as in (A)], and M (gray) for *K* = 0.6 as shown in (C).

The actual distribution of hydrophobicity in a protein is the result of hydrophobic inter-residual interactions. This distribution is called O (observed) later on in this paper.

These interactions, according to Levitt [31], are described by the function:

$$\begin{array}{c} \begin{array}{c} H_{i}^{O}=\frac{1}{H_{\mathrm{sum}}^{O}}\sum_{j}\\ \left\{\begin{array}{c} \left(H_{i}^{r}+H_{j}^{r}\right)\left(1-\frac{1}{2}\left(7{\left(\frac{r_{\mathrm{ij}}}{c}\right)}^{2}-9{\left(\frac{r_{\mathrm{ij}}}{c}\right)}^{4}+5{\left(\frac{r_{\mathrm{ij}}}{c}\right)}^{6}-{\left(\frac{r_{\mathrm{ij}}}{c}\right)}^{8}\right)\right)\;\text{for}\;r_{\mathrm{ij}}\leq c\\ 0,\;\text{for}\;r_{\mathrm{ij}}>c \end{array}\right. \end{array} \end{array}$$ (2)

where *r_ij_* is the distance between effective atoms (average position of atoms comprising a given amino acid) and *c* is the cutoff distance, which was set to 9 Ǻ according to [31]. *H^r^* denotes the intrinsic hydrophobicity of a given amino acid. Any scale of intrinsic hydrophobicity of amino acids may be used [32]. A comparison of both distributions, enabling the assessment of the degree of ordering of the hydrophobicity distribution in accordance with the reference distribution, is possible after normalizing both distributions [according to Eqs. ((1) and (2)—the first term in these formulas expresses normalization]. The example distribution (reduced to 1D presentation) is shown in Fig. 1A (pink line).

The assessment of the degree of similarity between the 2 distributions is expressed in the FOD-M model by Kullback–Leibler divergence entropy [33] according to Eq. (3):

$$D_{\mathrm{KL}}\left(P|Q\right)=\sum_{i=1}^{N}P_{i}\;{\text{log}}_{2}\frac{P_{i}}{Q_{i}}$$ (3)

where *P* represents the distribution in the protein under consideration—in the FOD-M model, this is the O distribution [Eq. (2)]. The Q distribution in Eq. (3) is a reference distribution—in the FOD-M model, this is the T distribution [Eq. ((1)].

However, the value determined using Eq. (3) cannot be interpreted directly (entropy). Therefore, a second reference distribution R was introduced, where each effective atom represents the same level of hydrophobicity *R*_i_ = 1/*N*, where *N* denotes the number of amino acids in the protein. The reference distribution T represents an arrangement with an idealized centric hydrophobic core. The reference distribution R represents an idealized arrangement completely devoid of any variation in hydrophobicity levels in the protein body.

A comparison of values with the relationship D_KL_(O|T) < D_KL_(O|R) indicates the similarity of distribution O to distribution T, which means the presence of a hydrophobic core in the analyzed protein. The inverse relationship expresses the absence of centralization of hydrophobicity in the protein body. In order to avoid using 2 values to describe a single object, the RD parameter was introduced:

$$\mathrm{RD}=\frac{D_{\mathrm{KL}}\left(O|T\right)}{D_{\mathrm{KL}}\left(O|T\right)+D_{\mathrm{KL}}\left(O|R\right)}$$ (4)

An RD value < 0.5 indicates the presence of a hydrophobic core (Fig. 1B).

The assumption of the presence of hydrophobicity distribution in the form expressed by the 3D Gaussian function has been justified. Numerous proteins representing structures with hydrophobicity distribution expressed by very low RD values have been identified. These are proteins from the fast-folding and ultra-fast-folding groups, also referred to as down-hill proteins and type II antifreeze proteins [34]. The results of protein folding simulations taking into account the water environment according to the FOD model were used in [35].

The generation of a hydrophobic core with a polar surface reflects the directionality of the protein folding process through the active participation of the water environment (Fig. 2A). However, water is not the only environment in which proteins take on their biologically active form. Membrane proteins for stabilization in a hydrophobic membrane environment expose hydrophobic residues, directing polar residues toward the center of the molecule (especially when acting as an ion channel). The optimal distribution for membrane proteins is therefore the opposite of the distribution expressed by the 3D Gaussian function:

$$T_{i}={\left(T_{\mathrm{MAX}}-T_{i}\right)}_{n}$$ (5)

where index *n* denotes normalization. The 3D Gaussian function spread on the protein body produces particular T distribution. This value depends on protein under consideration. The *T*_MAX_ in this distribution is used in Eq. (5) and later on in Eq. (6).

**Fig. 2.**
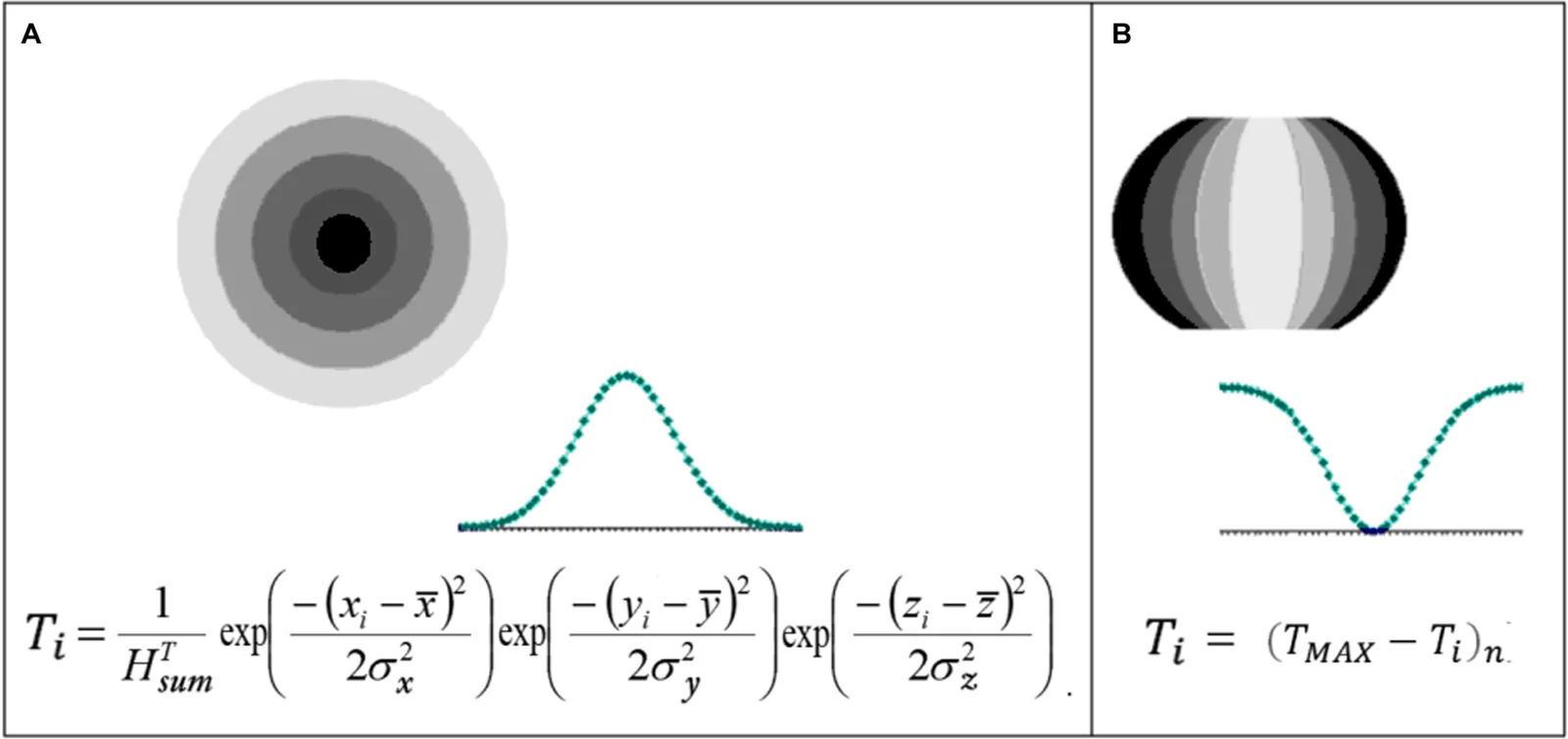
A diverse environment directs the folding process in a different way. (A) The influence of the water environment directing the folding toward the centralization of nonpolar groups (dark) and the exposure of polar residues (light)—the water environment. (B) The influence of the hydrophobic membrane environment leading to exposure of hydrophobic residues with centralization of polar residues. In both cases, the corresponding function expressing the given hydrophobicity distribution is given.

Both functions are illustrated in Fig. 2.

After analyzing numerous proteins [9] (including amyloids [6] and DNA binding [32]), the active participation of the environment in the folding process, which directs the distribution of amino acids in the protein body, takes the form of the sum of both proposed functions in the following form:

$$M_{i}={\left[T_{i}+K{\left(T_{\mathrm{MAX}}-T_{i}\right)}_{n}\right]}_{n}$$ (6)

The external field (M function-modified) expresses a combination of 2 different environments (3D Gaussian function and inverse function; Fig. 2) with a varied contribution of the inverse function expressed by the value of the *K* parameter. The value of *K* is calculated in iterative procedure as long as the minimum of D_KL_(O|M) is reached (Fig. 1C). The proteins listed above (down-hill, fast-folding, ultra-fast-folding, and some antifreeze proteins) [34], representing a distribution similar to a 3D Gaussian distribution, are described by the *K* parameter values = 0.0 or *K* < 0.3. Membrane proteins represent a hydrophobicity distribution with RD > 0.5, while *K* parameter values are mainly in the range of 0.6 to 1.3 [36].

The application of the discussed FOD-M model to the analysis of amyloid structures reveals significant changes in both RD and *K* values when comparing the native form of the protein with its amyloid variant [6], proving the need to change the external force field for amyloid transformation.

The procedure for obtaining the set of the RD and *K* parameters requires the following calculations:

1. determination of distribution T (Fig. 2A) according to Eq. (1)
2. determination of distribution O (Fig. 2A) according to Eq. (2)
3. determination of the values D_KL_(O|T) and D_KL_(O|R) according to Eq. (3)
4. calculation of the RD parameter value according to Eq. (4) (Fig. 2B). The value of the *K* parameter is determined in an iterative procedure by changing the value of the *K* parameter until the minimum value of D_KL_(O|M) is obtained (Fig. 1C) using function Eq. (5). It is calculated in iteration procedure starting from low value (it can be even 0.0), and then the values of D_KL_(O|M) are calculated with the step 0.1 for the *K* value. The M distribution is normalized for every *K* value (sum of all Mi values is equal to 1.0) before the D_KL_(O|M) is calculated.
5. the implementation of item 6 leads to obtaining the form of function M (Fig. 1D, gray line).

The significance of the M function determines the type of water environment modified by the contribution of a factor favoring an arrangement corresponding to the inverse 3D Gaussian function. Assuming that the folding polypeptide chain adapts the arrangement of amino acids to a position corresponding to the orientation imposed by the environment (exposure of polar residues in water or nonpolar residues in the membrane environment), the M function expresses the type of environment to which the folding chain adapts its structure. The M function, as a modification of the 3D Gaussian function through the introduction of a factor disturbing the specificity of the water medium, is the target for the protein, which during folding does not reproduce the 3D Gaussian function, but rather the M function. The M function represents the closest form to the T function by searching for the minimum value of D_KL_(O|M) (Fig. 2C). The M function acts as a modified (in relation to the water environment) target to which the folding protein adapts the distribution of amino acids depending on their intrinsic hydrophobicity.

The interpretation of the FOD-M model parameters is as follows:

1. The RD value determines the degree of reproduction of the micelle-like structure by the folded protein.
2. The value of the *K* parameter determines the degree of difference between the environment and the water environment in which the folding process took place.

The values of RD and *K* can be calculated for any protein using the programs in Open Access system as it is given in

The values of the RD and *K* parameters can be determined for a protein in the form of a single chain, but also for an object consisting of several proteins in a complex. The status of a single domain can also be assessed. In these cases, the 3D Gaussian function is generated individually for each unit.

It is also possible to determine the status of a given unit within a parent unit—e.g., a domain in a chain structure or a single chain in a complex. In this case, selected fragments of T and O profiles are normalized and the RD value is calculated according to Eq. (3) without assigning a separate 3D Gaussian function to selected fragments.

It is also possible to analyze the status of any selected fragment of the chain, e.g., by eliminating those residues whose Oi and Ti levels differ significantly. Such stepwise elimination of residues may lead to obtaining an RD value < 0.5, which allows the part of the protein chain obtained in this way to be assessed as representing a distribution consistent with a micelle-like arrangement, and thus, for example, the fragment responsible for the solubility of a given protein can be identified. Most often, the eliminated residues representing significant differences in Oi and Ti levels are catalytic residues. Generally, the difference between Ti and Oi levels is characterized by the relationship Ti > Oi, which indicates a local hydrophobicity deficit and most often concerns residues present in the cavity prepared for interaction with a ligand or substrate in the case of an enzyme. The relationship Oi > Ti indicates the exposure of hydrophobicity on the protein surface by a given residue, which may prepare for a possible hydrophobic interaction with another protein, leading to the formation of a complex.

The described FOD-M model is used in this study for a comparative analysis of human tubulins and the prokaryotic tubulin homolog FtsZ in order to reveal differences in hydrophobicity distributions and demonstrates the contribution of a specific external force field influencing the structure of a given protein prepared to perform its expected biological function.

External force field (*E*_EXT_) expressed by *K* is a term used to describe not only the environment of water or membranes but also the environment provided by different supporting proteins: prefoldin [37], chaperonins [38,39], and chaperones [40].

### Software

The program enabling calculation of RD is accessible on the GitHub platform: https://github.com/KatarzynaStapor/FODmodel and calculation of RD and *K* on the open access system https://hphob.sano.science platform.

## Results

### Comparative analysis of human tubulin and homologous bacterial protein

Table 1 summarizes the values of the RD and *K* parameters for the proteins being compared. There is a clear difference in the form of exceeding the discrimination level RD = 0.5, indicating a structure far from the micelle-like type in the case of eukaryotic protein and an RD value lower than the discrimination level for prokaryotic protein. Additionally, the differences in the *K* parameter values are significant. The prokaryotic protein clearly indicates orientation in the form of a water environment, while *K* = 0.6 for the β chain of tubulin indicates the need for an external orienting factor in the folding process.

**Table 1.** The RD and *K* parameter values describing the status of the proteins in question and the domains present in them. The values given here describe the status of the listed structural units treated as individual units—this means generating a 3D Gaussian function for each unit individually.

PDB ID	Fragment	RD	*K*
1FSZ	Chain	0.454	0.3
	D1 (38–249)	0.355	0.2
	D2 (250–355)	0.351	0.2
6I2I	Chain B	0.572	0.6
	D1 (1–261)	0.612	0.6
	D2 (262–383)	0.502	0.4
	D3 (384–436)	0.605	0.6

The visualization of the hydrophobicity distribution in the form of T, O, and M profiles for the FtsZ protein reveals a highly consistent observed distribution O compared to the idealized distribution T in the form of a 3D Gaussian function. The value *K* = 0.3 represents a slight modification of the 3D Gaussian function, which suggests that the folding process is directed in accordance with the micelle-like structure.

The independent, spontaneous folding of the FtsZ protein chain is further supported by the independent folding of domains whose status is described by even lower RD and *K* values (Table 1). Each domain exhibits an individual hydrophobic core, whose appearance during the folding process significantly facilitates its course and enables spontaneous folding in the water environment.

By carefully analyzing the T and O profile relationships, it is possible to clearly identify the location of the hydrophobic core, where the expected Ti level is almost exactly reproduced in the actual distribution in the Oi form. There is also a slight excess of hydrophobicity visible on the surface, where Oi levels exceed the expected Ti levels. These fragments are marked with a red line on the horizontal axis of the profile set and with red color in the 3D presentations (Fig. 3). Based on the FOD-M model, the role of these fragments can be interpreted as being prepared for interaction with other molecules in order to form the fibril-like structure expected for the predicted function of this protein.

**Fig. 3.**
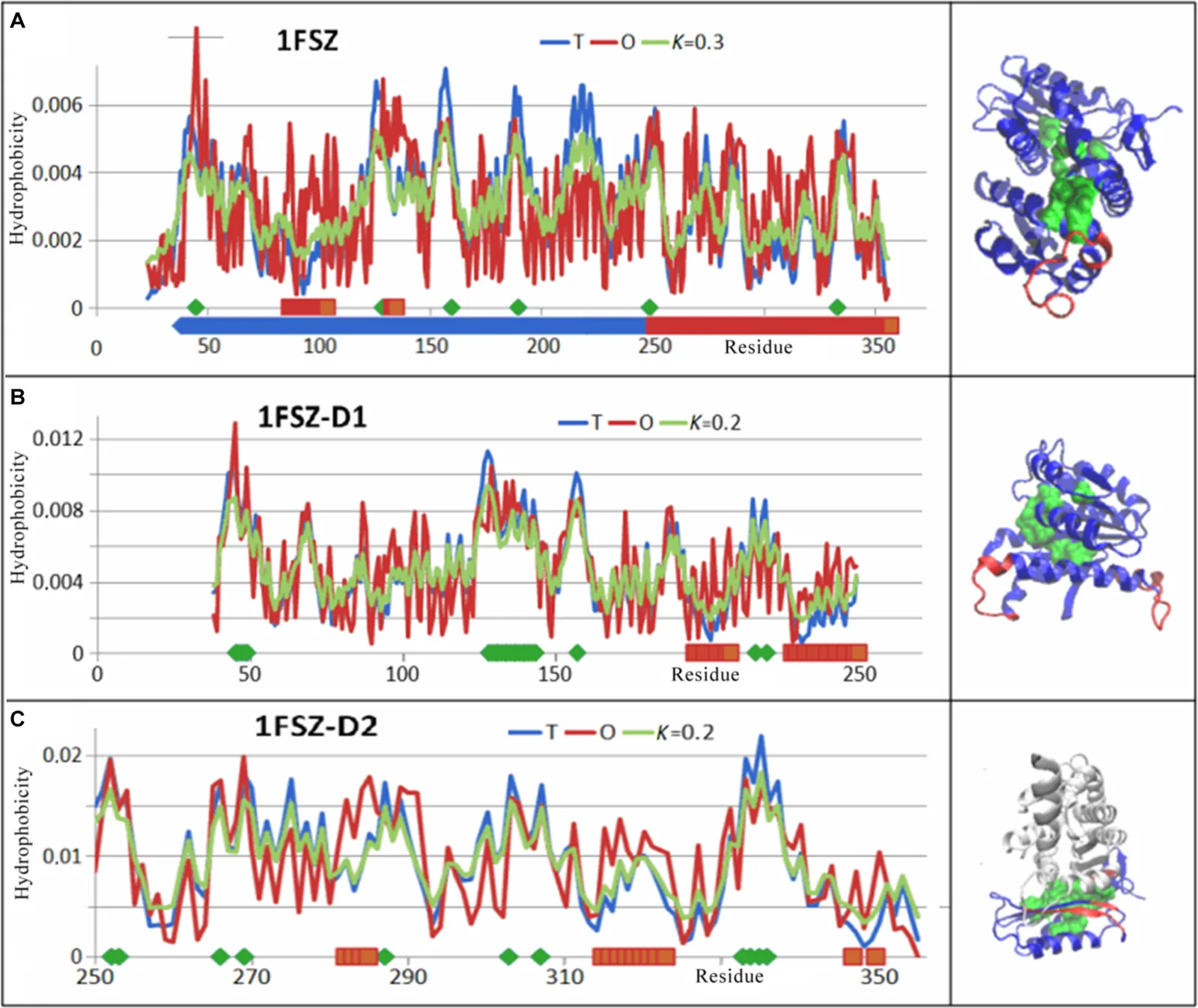
T (blue), O (red), and M (green) profiles for *K* values given in captions. (A) Complete FtsZ protein chain (bottom blue/red line, domains). (B) Status of domain D1. (C) Status of domain D2. Relevant 3D structure presentations were also provided. The points on the lower axis: red positions, excess hydrophobicity; green, local maximum of hydrophobicity. A similar distinction in 3D presentations. Green, hydrophobic core; red, hydrophobic exposure—potential site for complexing another chain.

Profiles T, O, and M for human tubulin β-chain visualize lower adaptation of profile O to profile T, as indicated by the higher RD parameter value (RD > 0.5). The expected high levels of hydrophobicity to generate a hydrophobic core are not represented by the corresponding Oi levels. The local excessiveness of hydrophobicity levels is also significantly higher, e.g., visible in the region (100 to 120). The evaluation of the comparison of distribution O with distribution T suggests the absence of a clearly defined hydrophobic core in β chain of tubulin. The value of the *K* parameter is also higher, indicating the need to introduce an external force field different from the water environment for the folding of chain B of human tubulin.

Additionally, the status of the domains also shows a low match between the O distribution and the T distribution, suggesting a mismatch with the orientation resulting from the presence of the water environment. Unlike the domains of the FtsZ protein, none of the domains present in chain B of human tubulin obtain their structure as a result of active participation of the water environment.

The results of the evaluation of the structures of the compared proteins given in Table 1 and Figs. 3 and 4 explain the reasons for the differences in the characteristics of the folding process. The folding of the bacterial protein FtsZ (a homolog of tubulin) occurs spontaneously under the influence of the water environment. The folding of tubulin indicates the need for the presence of and cooperation with an external factor other than water. The supplier of the correct external force field is chaperonin TRiC/CCT. The value of the *K* parameter quantitatively determines the need for an external force field with a specificity expressed by the value *K* = 0.6 [Eq. (6)].

**Fig. 4.**
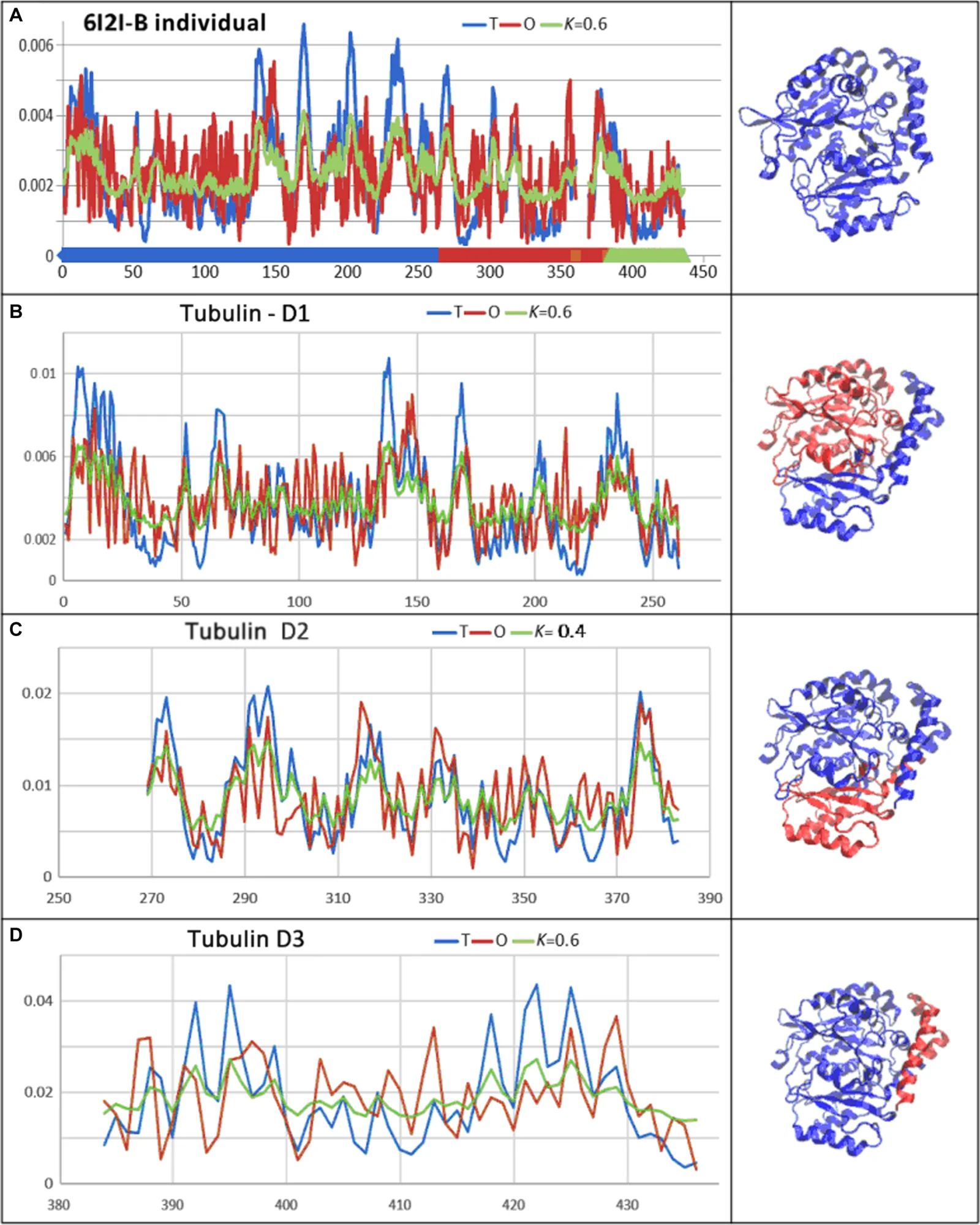
Profiles T (blue), O (red), and M (green) for *K* values given in the captions determining the status. (A) T, O, and M distributions for chain B human tubulin—domain ranges are marked on the horizontal axis. (B) Status of domain D1. (C) Status of domain D2. (D) Status of domain D3 [green line in (A)], along with a 3D presentation, where the relevant domains were highlighted in color (red).

The M profiles for the compared proteins show differences in the form of a minimally different profile T in the case of the FtsZ protein. The M distribution profile shows significantly smaller deviations, which indicates a rather flattened distribution of hydrophobicity within the protein body.

The proteins being compared differ in chain length. In search of local similarities in hydrophobicity distributions, T profiles of both proteins were superimposed (Fig. 5A) in a manner that reproduces the shape of the T profiles for both compared proteins as closely as possible taking the FOD-M-based criteria. The positions of local maxima are overlapped. Each maximum represents the residue localized in the central part of the molecule. This is why the overlapping reconstructs the common potential hydrophobic core in both proteins.

**Fig. 5.**
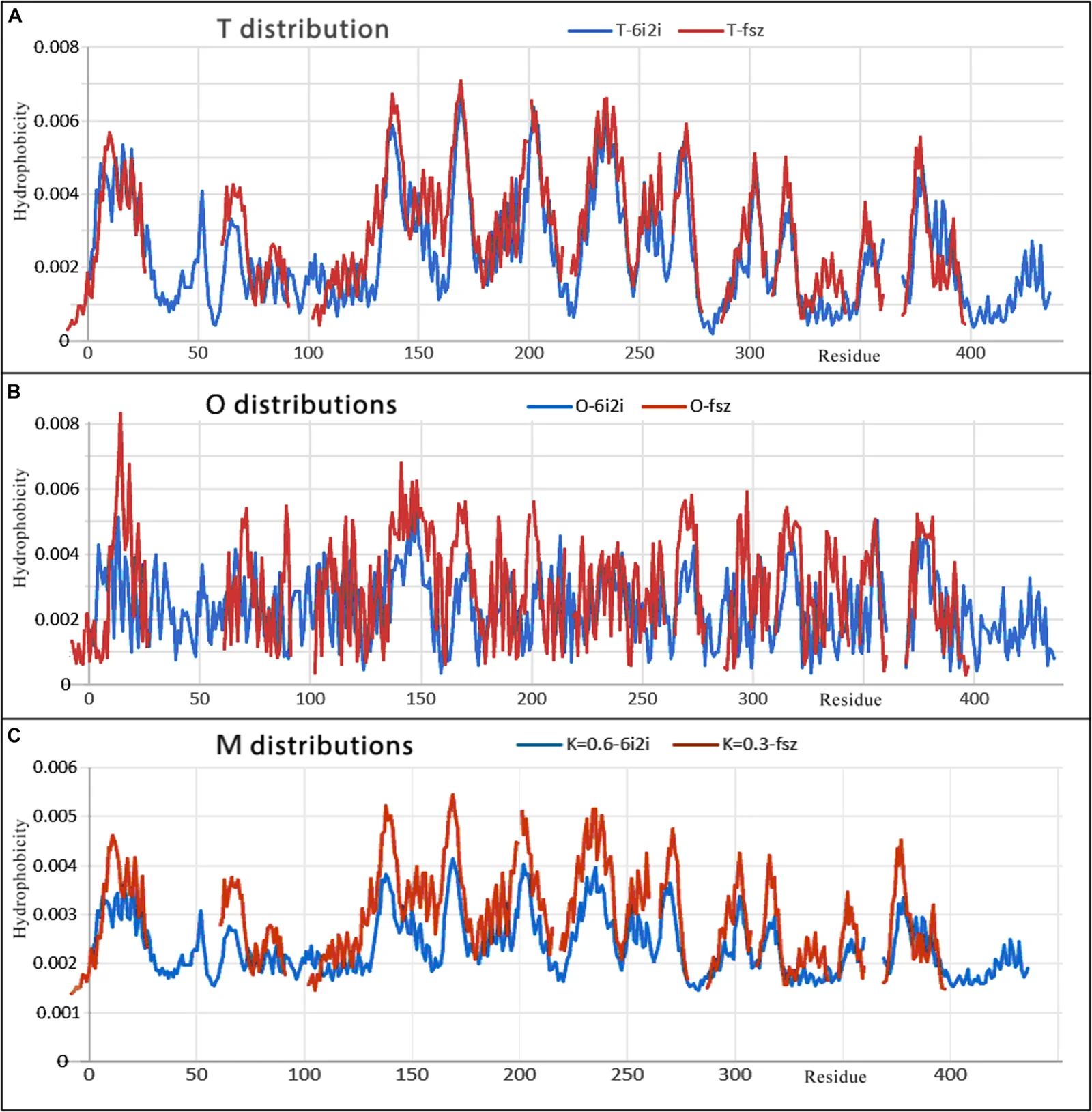
The summary of the distribution profiles of the compared proteins, assuming maximum similarity of the positions of local maxima and minima. (A) T distributions for tubulin (blue) and FtsZ (red)—correlation coefficient 0.814. (B) O distributions for tubulin (blue) and FtsZ (red) when superimposed as achieved for T distributions—correlation coefficient = 0.297. (C) M distributions for tubulin (blue) and FtsZ (red) when superimposed as achieved for T distributions—correlation coefficient = 0.799.

The correlation coefficients of 0.814 (Fig. 5) suggest high similarity of the selected T-profile fragments. The same superimposition of the O distributions already shows a significantly lower correlation coefficient (0.297), which indicates a different distribution of hydrophobicity in the actual protein structure despite the similarity of the T distributions. The M distributions—as directly dependent on the T distributions, although determined for a different *K* value—show high consistency (0.799).

Interpretation of this part of the results indicates a very similar idealized distribution for both proteins—similar target distributions (T profile) to which the folding protein attempts to adapt. A very low similarity between the O distributions means that the proteins have adapted to a different degree to the target, which is assumed to be the T distribution. The M distribution is a distribution of a directly modified 3D Gaussian function, hence the high correlation coefficient (0.799). Different O distributions indicate a different degree of discordance with respect to external conditions imposed by the water environment. This discordance in adapting to the external force field in the case of tubulin stems from the fact that other external conditions are imposed for the folding of this protein. Therefore, the correlation coefficient for comparing M distributions is lower than that for comparing T distributions.

The analysis of the degree of similarity of distributions for analogous fragments of chains was obtained after eliminating those fragments that did not find their analogs in the distribution of the second protein (this mainly applies to tubulin due to its longer chain) (Fig. 5).

Figure 6A reveals segment absent in the FtsZ protein. Figure 6B assesses the status of these eliminated fragments in tubulin as highly mismatching the T distribution for this protein. It can therefore be concluded that the inserted region in tubulin is the cause of the mismatch between the T distribution and the O distribution present throughout the molecule. Based on the high value of RD = 0.591 and *K* = 0.8, the role of these segments can be interpreted in this way.

**Fig. 6.**
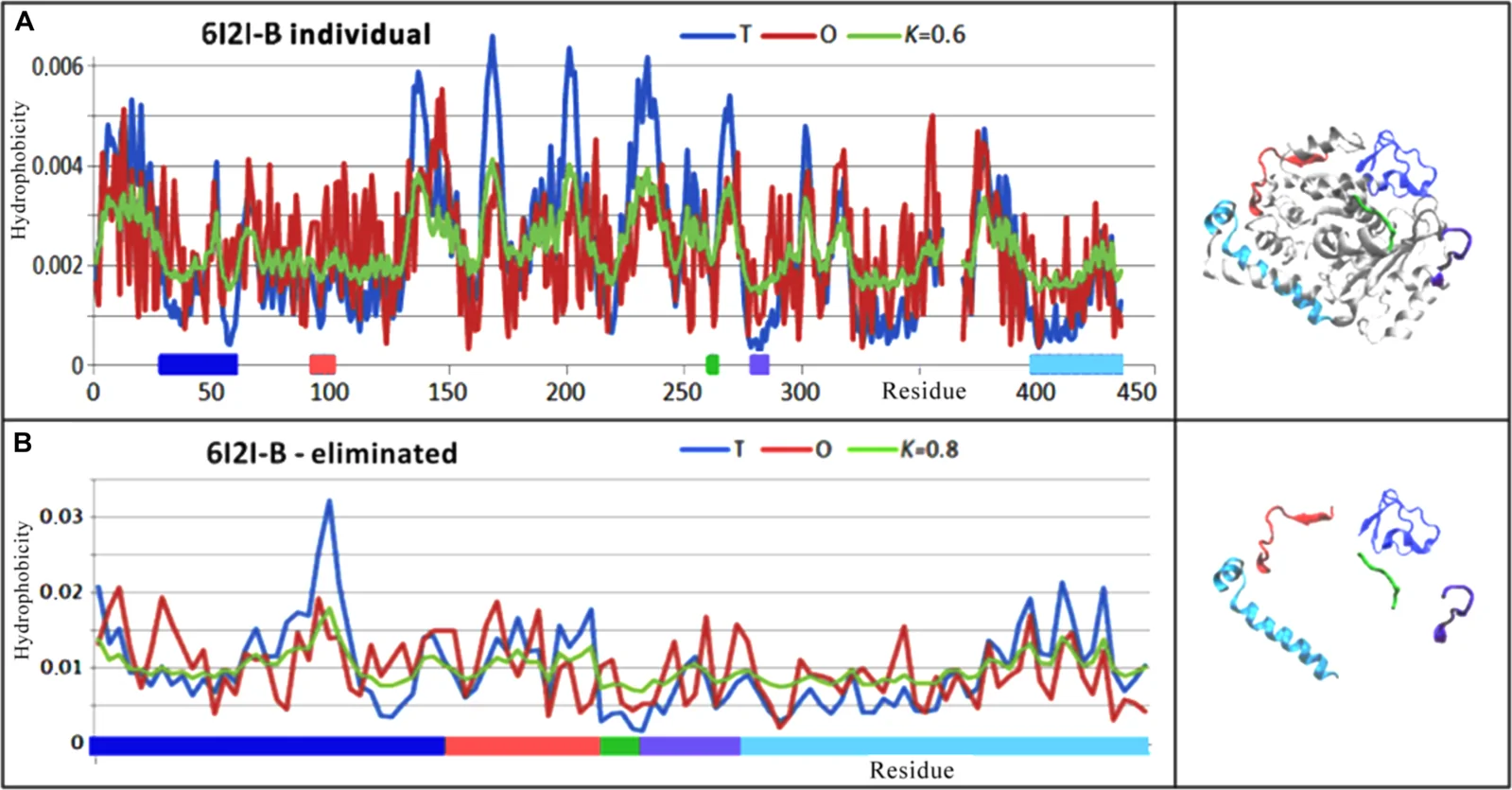
Elimination of fragments differentiating profiles for tubulin and FtsZ. (A) T (blue), O (red), and M (green) profiles for chain B tubulin with highlighted fragments that were eliminated in the procedure of superimposing the FtsZ and tubulin protein profiles (Fig. 5). (B) Status expressed by T, O, and M profiles for the assembled segments eliminated in the procedure of superimposing the FtsZ protein profile. On the horizontal axis, the relevant segments are highlighted in color and are also presented in 3D as components of the tubulin structure (top) and highlighted separately (bottom). Blue, (28 to 58); red, (94 to 101); green, (261 to 264); violet, (280 to 285) cyan, (397 to 435)—numbering system according to sequence of β chain of tubulin (6I2I).

Figure 6B reveals the locations of eliminated segments that are comparable to those identified in [24] despite different criteria for identifying differences (Fig. 6).

The status of parts of chains lacking fragments that do not have counterparts in the other protein (Table 2) indicates that despite the similar position of the expected local maxima, these are not achieved in tubulin. It can be speculated that in the water environment, it is possible to obtain a tubulin structure with a higher proportion of micelle-like forms. To prevent this, the synthesized tubulin chain is transported with the help of prefoldin, preventing micelle-like structuring. The increased value of *K* = 0.6 for tubulin is the result of the external force field obtained through isolation from the water environment by prefoldin and ATP-dependent ring-shaped chaperonin TRiC/CCT.

**Table 2.** RD and *K* parameter values for parts of fragments with a high degree of similarity of T distributions (as shown in Fig. 5)

Fragment	RD	*K*
Human	0.541	0.5
Bacterial	0.460	0.3

Table 2 shows the parameter values for parts of analogous chains (after eliminating segments that have no analogs in the form of T, O, and M distribution). The variability remains similar to the comparative analysis of complete chains.

Reduced stability in the absence of a clearly defined hydrophobic core with a specific constant exposure for complexation purposes would lead to a system that is too rigid, inflexible, and resistant to change. The relatively unstable structure of human tubulin reveals a general strategy leading to the final effect in the form of a chain with adapted specificity related to the expected function. Tubulin molecules that form microtubules—components of the cytoskeleton—are expected to exhibit dynamic instability that allows for assembly and disassembly. Both of these processes are based on reversible self-organizing. To achieve this goal, structural plasticity is needed, which is re-organizing itself continuously.

The folding of tubulin into micelle-like forms with a rigid hydrophobic core and constant, clear-cut exposure of hydrophobicity for the purpose of complex formation would place too great a restriction on the required flexibility of the cytoskeleton [41–44].

Depending on the expected function performed by a given protein, there are examples of strong stabilization in the form of a clearly defined hydrophobic core. This applies to the stabilization of the domain present in dystrophin [45,46] and titin—a component of muscle tissue [47]. These are examples of proteins that are exposed to considerable physical stress as part of their function. Protection against structural deformation and the guarantee of a return to the original form is achieved by the presence of a clearly defined hydrophobic core, which can be restored in the event of deformation.

To make the analysis representative, the set of human tubulins in different structural forms (forms of complexes) was analyzed. The mean values of RD and *K* are given in Table 3.

**Table 3.** The mean values of RD and *K* calculated for the set of human tubulin β chains representing the different organization of complexes

Form	RD	*K*	Number of examples
Dimer (α + β)	0.572	0.6	1—6I2I (β)
Linear	0.561	0.583	34
2D	0.583	0.611	59
All	0.574	0.59	94

The RD and *K* of tubulin analyzed in the paper (6I2I) appear very similar to averaged values of those representing the set of tubulins (β).

## Discussion

The research presented in [23] discusses in detail the stages of the tubulin folding process with the active participation of TRiC/CCT chaperonins, distinguishing 4 stages of structural changes leading to the biologically active form. The role of prefoldin is also interpreted. These stages are the subject of a separate analysis based on an interpretation using the FOD-M model.

The folding process of tubulin and actin involving chaperone proteins clearly indicates the need to introduce variability in external conditions in addition to sequence variability. This spectrum of variability of external conditions turns out to be very broad. The structural specificity of Hsp proteins provides a huge range of variability in environmental conditions. Consequently, it is proposed that the folding process be treated as the result of multi-object optimization. In the process of predicting protein structures using *in silico* methods, the optimization process should take into account both the optimization of the internal force field (nonbinding interatomic interactions within the protein body) and optimization from the point of view of environmental preferences—the external force field [Eq. (7)]. The function expressing the energy state of protein E should take the following form:

$$E=E\left(E_{\mathrm{INT}},{\;E}_{\mathrm{EXT}}\right)$$ (7)

Based on the FOD-M model, *E*_EXT_ should depend on the *K* parameter characterizing the specific nature of the environment.

In previous studies, the scale of the *K* parameter value ranged from 0 to 10, where *K* < 0.4 represents a water environment, and *K* values in the range 0.5 to 1.3 mainly represent a membrane environment. However, the *K* value range 0.6 to 10.0 corresponds to the environment introduced by chaperonins [37–40].

In practice, multi-object optimization requires the use of a method called the Pareto front [48,49]. This procedure leads to a solution in the form of a consensus between optimization resulting from interactions within the protein body (*E*_INT_) and environmental influences (*E*_EXT_).

The 2 examples discussed here, which differ in terms of the nature of the folding process, illustrate the need to apply the multi-object optimization procedure when attempting to simulate the protein folding process using *in silico* methods.

The protein folding problem is presented as unknown search for proper energy minimum representing the stable structure. The search for unknown solution is visualized in traditional form as shown in Fig. 7A. The hypothesis based on the FOD-M model suggests that the proper energy minimum can be found using the directional role of external force field (Fig. 7B). This is why the qualitative horizontal axis (Fig. 7A) is proposed to be substituted by quantitative scale of *K* values pointing the proper external conditions directing folding process toward the expected local or global energy minimum.

**Fig. 7.**
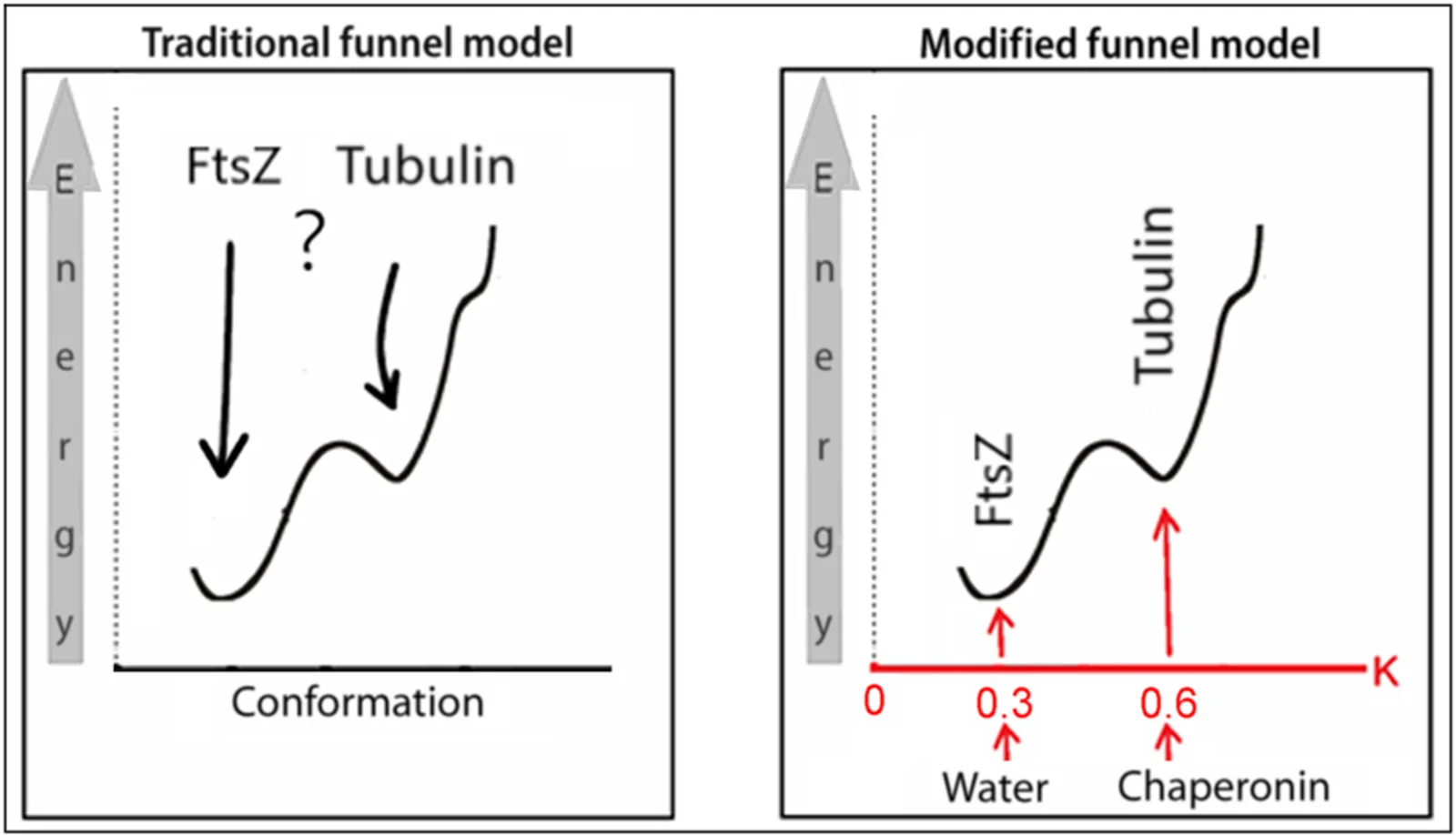
The funnel model in its traditional form (left side) and modified form (right side), where the choice of the appropriate local energy minimum is dictated by the external force field. Hypothesis based on the presented analysis.

Consequently, a modified funnel model is proposed (Fig. 7), where obtaining a structure corresponding to the local energy minimum is the result of the participation of an environment with different characteristics expressed by the value of the *K* parameter [6].

The problem to be solved is the relation between different physical mechanisms that may generate similar *K* values. The main factor determining the 3D structure is the sequence of amino acids in polypeptide chain. The same sequence, however, may adopt different structure depending on environment. The amyloids are proteins with no mutation inserted adopt entirely different structure. The assumption is that it is the effect of environmental changes.

The suggestion based on FOD-M model analysis is that the same amino acid sequence may adopt different structures described by different *K* values. Different sequences may reach the structures described by equal *K* value. The problem is how certain amino acid sequence searching for optimal structure (low *E*_INT_) reacts on the surrounding. The example of unfolding–refolding the proteins in environment changed by the presence of urea is a good example for the discussed problem.

To characterize the FOD-M model, some aspects will be pointed out:

1. The model is of descriptive character despite the quantitative parameters RD and *K* since it does not enter into the mechanism of the folding process
2. The value of the *K* parameter may be recognized for many different sequences, and the same amino acid sequence may generate structures described by the same *K* value. This is why the *K* parameter may not be treated as deterministic one.
3. The correlation coefficient characterizing the structures of FtsZ and human tubulin β chain is based on subjective examination of structure overlapping using the localizations of local maxima in T distribution as the criteria.
4. The presented results are of preliminary character. Generally, the larger set of human β-chain tubulins will be analyzed.

## Conclusions

A comparative analysis of homologous proteins performing the same function—cytoskeletal structures—in the prokaryotic organism FtsZ and the eukaryotic organism human tubulin chain B, conducted on the basis of the FOD-M model, reveals fundamental differences in the structure of these 2 proteins. It has been demonstrated that in order to obtain a biologically active form through a prokaryotic protein, the environment directing the folding process is a water environment conducive to the formation of a centrally located hydrophobic core and a polar shell. Human tubulin requires the active participation of both prefoldin and the chaperonin TRiC/CCT in the folding process. The process carried out under such conditions leads to the protein acquiring a structure with a blurred hydrophobic distribution, lacking a clearly defined hydrophobic core. This status is linked to the need for tubulin and actin to reveal multiple structural states, which significantly affect its ability to polymerize and depolymerize, which is related to its biological function.

The folding of FtsZ based on the presence of a specific hydrophobic core represents an example of a protein whose folding does not require the participation of other supporting proteins, as the structure of this protein forms spontaneously with the active participation of only the water environment.

## Data Availability

The program enabling the calculation of RD is accessible on the GitHub platform: https://github.com/KatarzynaStapor/FODmodel and calculation of RD and *K* on the open access system https://hphob.sano.science platform. The output file (Excel) delivered by HPHOB SANO presents the profiles: T, column C; O, column D; M, column O. The RD and *K* values are available in separate output file (.txt).
